# Heat Transfer Fluids as Co‐Diluents in Localized High‐Concentration Electrolytes for High‐Rate Lithium Metal Batteries With Enhanced Safety

**DOI:** 10.1002/anie.3797296

**Published:** 2026-06-16

**Authors:** Dominik Weintz, Adil Aboobacker, Andrew Dopilka, Felix Pfeiffer, Anne Hockmann, Uta Rodehorst, Christian Wölke, Monika Schönhoff, Robert Kostecki, Diddo Diddens, Martin Winter, Isidora Cekic‐Laskovic

**Affiliations:** ^1^ Helmholtz‐Institute Münster (IMD‐4) Forschungszentrum Jülich GmbH Münster Germany; ^2^ Energy Technologies and Systems Division Lawrence Berkeley National Laboratory Berkeley California USA; ^3^ Institute of Physical Chemistry University of Münster Münster Germany; ^4^ International Graduate School For Battery Chemistry, Characterization, Analysis, Recycling, and Application (BACCARA) University of Münster Münster Germany; ^5^ MEET Battery Research Center University of Münster Münster Germany

**Keywords:** electrolyte diluent, electrolyte transport kinetics, ion clustering, lithium metal battery, localized high‐concentration electrolyte

## Abstract

Localized high‐concentration electrolytes (LHCEs) have been identified as promising electrolyte formulations for lithium metal batteries, due to their effective interphase formation and promotion of compact Li deposition, yet their practical implementation is often limited by reduced ion transport kinetics. In this study, two industrially established fluorinated ethers are identified for the first time in battery research as effective co‐diluents as they combine a broad electrochemical stability window with a low viscosity and intrinsic non‐flammability. Incorporating these components, commonly used as heat transfer fluids, yields safer, less flammable electrolyte formulations with enhanced ion mobilities. In particular, the ternary co‐diluent formulation shows improved ion mobility by reducing the electrolyte's viscosity while limiting excessive ion clustering. Based on the improved electrolyte transport kinetics, lower overvoltages and higher Coulombic efficiencies at current densities ≥ 1 mA cm^−2^ are achieved with the ternary co‐diluent blend, resulting in markedly extended cycle life in an application‐oriented zero‐excess pouch cell compared with the baseline system. Complementary electrochemical and ex situ analysis of harvested electrodes at moderate current densities reveals no discernible differences in interphase morphology and composition, suggesting enhanced ion mobility as the primary cause of the improved high‐rate performance.

## Graphical abstract

Two fluorinated ethers, commonly used as heat‐transfer fluids, are explored as co‐diluents in localized high‐concentration electrolytes (LHCEs) for lithium metal‐based batteries. The best‐performing ternary diluent blend boosts ion mobility and cuts overvoltages, thereby improving Coulombic efficiency and cycle life at ≥1 mA cm^−2^ in zero‐excess pouch cells, which is attributed to a reduced viscosity and suppression of extensive ion clustering.

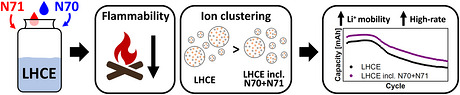

## 1 Introduction

The increasing deployment of portable devices and electric vehicles, combined with recent advances in electrolyte engineering, has led to the revival of the lithium metal battery (LMB) as a viable electrochemical energy storage technology [1, 2, 3, 4, 5, 6]. Nonetheless, the challenges of implementing a metallic lithium electrode that plagued researchers for decades remain [2, 4, 7]. In addition to safety hazards, ineffective Li metal protection and inhomogeneous Li deposition promote dead Li formation and lead to rapid liquid electrolyte consumption and depletion of the Li inventory, lowering the Coulombic efficiency (CE) and limiting the cycle life of the resulting cells [7, 8]. The electrolyte plays a crucial role in governing the composition, structure, and formation of the solid electrolyte interphase (SEI) and is therefore a key lever in addressing the challenges hindering broad LMB commercialization [2, 9, 10, 11, 12, 13]. With the latest advancements in material science and targeted synthesis of solvent molecules, considerable progress toward the desired 99.9 % CE has been made [2, 4, 14, 15, 16, 17, 18]. Among recent research approaches, localized high‐concentration electrolytes (LHCEs) have emerged as a promising electrolyte concept by combining the favorable solvation structure and interfacial chemistry of high‐concentration electrolytes (HCEs) with the wetting properties and cost structure of dilute systems [19, 20, 21]. The enhanced anion coordination in LHCEs promotes an effective inorganic SEI and a compact Li deposition morphology, enabling high CEs and extended lifetime of LMBs [22]. The diluent plays a unique role in determining vital electrolyte properties, depending on its viscosity and interactions with the inner Li solvation shell. It is designed to show minimal to no Li^+^ ion solvation, leaving the primary solvation sheath intact and essentially occupying the volume between the salt‐solvent clusters without disrupting the formed contact ion pairs (CIPs) and aggregates (AGGs) of the HCE analogue [23]. Consequently, the diluent must meet a demanding set of properties, including a low donor number and low solvation of Li^+^ ions, a sufficient miscibility with the concentrated electrolyte, and an electrochemical inertness over the cells' operating voltage range [19, 24]. For practical LMB operation, it should further exhibit low viscosity for improved wetting and ion transport, reduced flammability, and acceptable environmental and cost profiles [25]. Especially fluorinated ethers such as 1,1,2,2‐tetrafluoroethyl 2,2,3,3‐tetrafluoropropylether (TTE) [20, 24, 26] and bis(2,2,2‐trifluoroethyl)ether (BTFE) [27, 28, 29] have been identified as suitable candidates to meet the requirements [30, 31]. The incorporation of fluorine substituents lowers both the donor number and dielectric constant based on their electron‐withdrawing character, thereby minimizing the Li^+^ ion coordination, whereas the ether group improves miscibility with the ether‐based HCE matrices. Fluorination also generally leads to lower HOMO energy levels, enhancing stability (typically >4.2 V vs. LiǀLi^+^, depending on formulation) and mitigating decomposition reactions in LMBs with common positive electrodes (e.g., LiNi_0.8_Mn_0.1_Co_0.1_O_2_ (NMC811)) [32]. As a result, fluorinated ether‐diluted LHCEs have been shown to produce robust, inorganic‐rich interphases and enable efficient Li plating/stripping, even outperforming their analogous HCEs [19, 20, 33, 34]. However, despite their widespread use, common diluents pose several limitations for LHCEs in practical LMBs. While the addition of diluents typically reduces the viscosity of the LHCE without disrupting the anion‐rich coordination environment, improvements in ionic conductivity are often only marginal, thereby limiting high‐rate operation. Moreover, a limited understanding of how micro‐ and mesoscopic solvation structures govern mass transport and interfacial reactivity impedes targeted electrolyte design [18, 19, 31].

**FIGURE 1:**
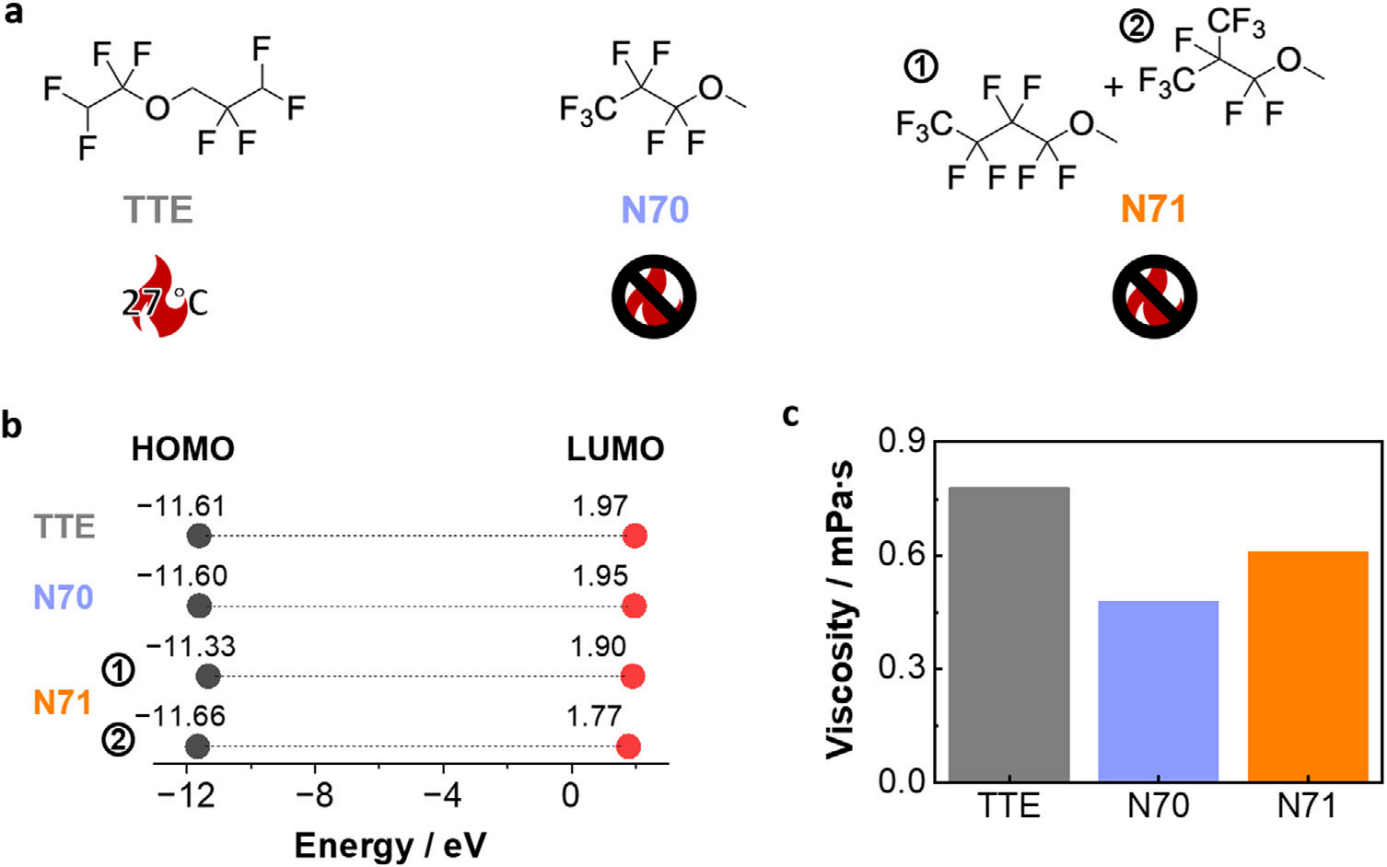
(a) Molecular structures and flash points of 1,1,2,2‐tetrafluoroethyl 2,2,3,3‐tetrafluoropropylether (TTE), methyl heptafluoropropylether (N70), and methyl nonafluorobutylether (N71). (b) HOMO/LUMO levels of the investigated diluents determined via DFT calculations, and (c) their dynamic viscosities at 20°C.

In this study, two fluorinated ethers, namely methyl heptafluoropropylether (N70) and methyl nonafluorobutylether (N71) (structures shown in Figure 1a), commonly used as low‐temperature heat transfer fluids, are investigated as potential (co‐)diluents for LHCEs, and their impact on the solvation structure and key electrolyte properties is evaluated [35]. These compounds are non‐flammable and possess low viscosity and dielectric constants, preventing excessive Li^+^ ion coordination. In addition, their established supply chain and industrial use lead to lower production costs than those of many fluorinated ether analogs. By adding these diluents to the baseline electrolyte (LiFSI in DME/TTE (1:1.2:3 n/n)), consisting of lithium bis(fluorosulfonyl)imide (LiFSI), dissolved in dimethoxyethane (DME) and diluted by TTE, several key properties of the corresponding electrolyte formulation can be improved, including the flammability and ion mobility. Notably, the ternary co‐diluent blend comprising N70, N71, and TTE exhibits the most enhanced electrolyte transport kinetics due to limited ion clustering, thereby enabling markedly improved overvoltages, CEs, and cycle life at high current densities (≥1 mA cm^−2^) compared with the baseline system. Additional ex situ analysis reveals that the formed interphases appear identical after galvanostatic cycling at moderate current densities, suggesting that improved Li^+^ ion transport within the bulk electrolyte, rather than interphase modifications, is the sole cause of the enhanced high‐rate performance.

## 2 Results and Discussion

### 2.1 Physicochemical Properties of the Diluent Candidates

To qualify as suitable diluents in LHCEs, the investigated fluorinated ethers N70 and N71 must meet several requirements. As the diluent makes up the majority of an LHCE, its flammability is a primary safety consideration. Both N70 and N71 are classified as non‐flammable and exhibit no measurable flash point. Their high fluorination suppresses radical chain propagation, resulting in enhanced flame‐retardant properties relative to TTE, which is flammable and has a flash point of 27°C (Figure 1a) [36, 37, 38, 39]. In application‐oriented cells (e.g., pouch cells), this reduces the diluent's contribution to thermal runaway, lowers the inventory of flammable components, and improves overall cell safety. In addition, their established synthesis and long‐standing use as heat transfer and precision‐cleaning fluids have enabled a reliable supply at high purities at competitive costs relative to many other fluorinated ether analogues.

Since the diluent should not directly interfere with the anion‐derived interphase formation, a broad ESW of the diluent is essential to prevent reductive and oxidative decomposition during cell operation. Density functional theory (DFT) calculations showed that the energy levels of the highest occupied molecular orbital (HOMO) and the lowest unoccupied molecular orbital (LUMO) differ by no more than 0.2 eV from those of TTE, suggesting comparable redox stability (Figure 1b). Even in the absence of strong intermolecular interactions, unexpected solvation and interfacial effects may affect reaction behavior within the electrolyte formulation. N71 and particularly N70 have markedly lower viscosities compared to TTE, which likely translates to improved viscosity and wetting properties of the corresponding LHCE (Figure 1c). From a practical perspective, N70 provides the largest viscosity reduction, but its low boiling point increases the risk of evaporation losses during manufacturing. While N71 has a higher viscosity than N70, its lower volatility simplifies handling during electrolyte formulation.

Collectively, the non‐flammability, broad ESW, and low viscosity, combined with assumed weak Li^+^ ion solvation, make both N70 and N71 suitable candidates as diluents in LHCEs.

### 2.2 Electrolyte Properties

**FIGURE 2:**
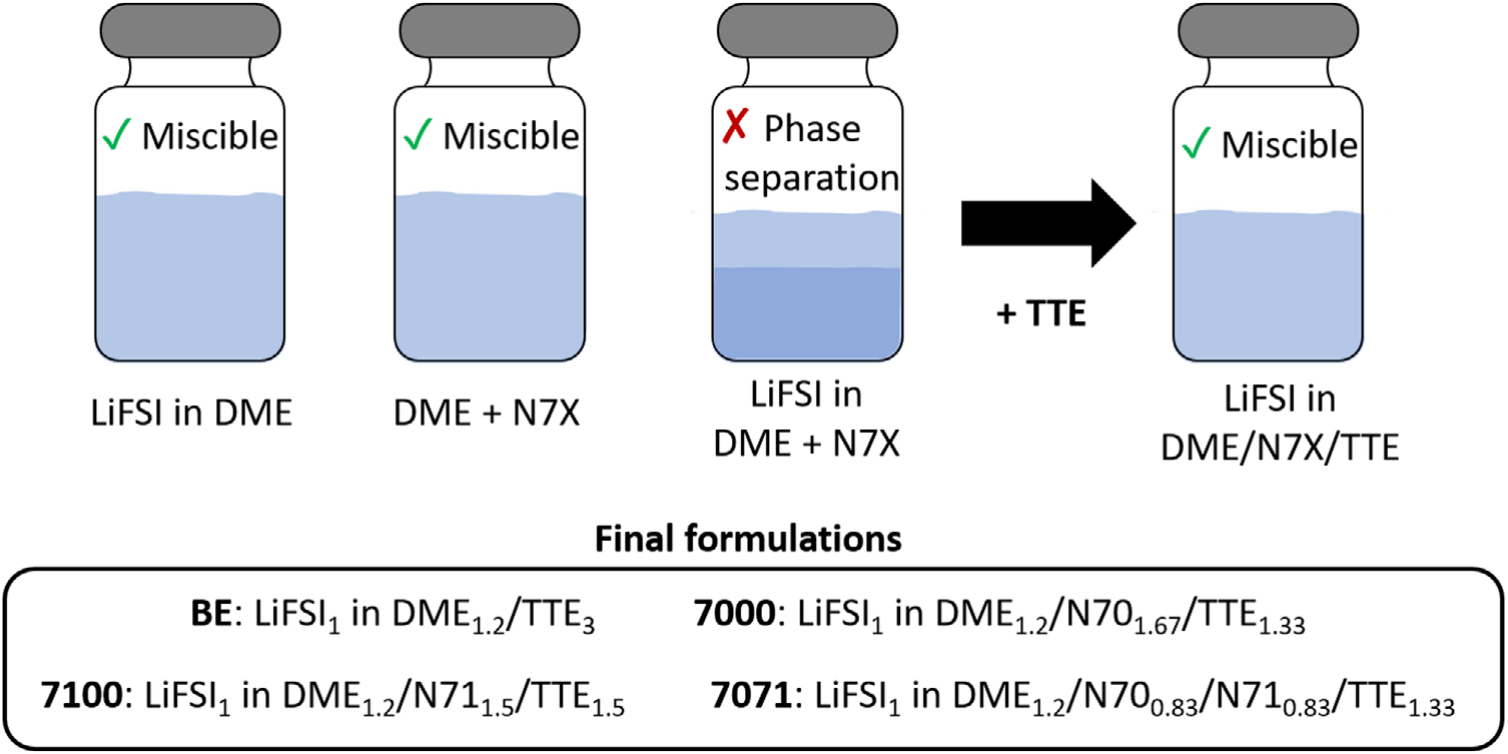
Phase‐behavior schematics with N70 and N71, demonstrating the need for TTE as a co‐diluent to achieve a homogeneous solution (top). Considered electrolyte formulations used for this study with the respective molar ratios of the components (bottom). N7X refers to N70 and/or N71.

The fluorinated compounds N70 and N71 show good miscibility with the solvent DME, however, upon addition of the conducting salt, a macroscopic phase separation occurs. For this reason, TTE was added as a co‐diluent to the electrolyte, serving as a miscibility agent to facilitate the merging of the two phases into a macroscopically homogeneous solution. As a result, three liquid electrolytes were formulated with the goal of maximizing the N7X/TTE ratio (N7X ≙ N70 and/or N71): two comprise each of the diluents, and a third combines both in a single system (Figure 2).

**FIGURE 3:**
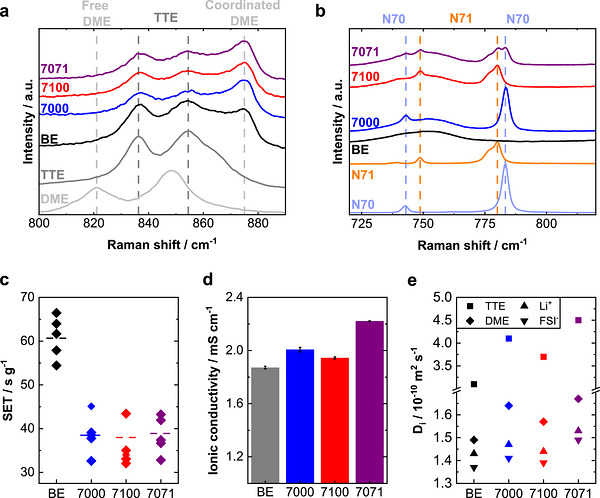
Raman spectra of the considered liquid electrolyte formulations, illustrating how the addition of N70 and N71 impacts the solvents' characteristic vibration bands and thus the intermolecular interactions of (a) TTE, DME, and (b) N70, N71. (c) Flammability tests of the considered electrolyte formulations demonstrate the impact of N70 and N71 on the flammability of the respective electrolyte. (d) Ionic conductivity of the considered electrolyte formulation and (e) diffusion coefficients of selected electrolyte components dependent on the electrolyte chemistry at 20°C.

Complementary analysis of the solvation structure via Raman spectroscopy indicates that adding the weakly solvating fluorinated ethers has a low impact on the solvation structure and intermolecular interactions. Like in the baseline LHCE (BE), no free DME molecules are present as they are fully coordinated in the Li^+^ and FSI^−^ ion clusters. The Raman bands at 875 cm^−1^, associated with coordinated DME, remain constant in both position and overall shape across the investigated electrolyte formulations, indicating a similar primary ion solvation structure (Figure 3a) [40]. The coordination of the TTE also remains consistent across all considered electrolytes, with the Raman band at 836 cm^−1^ exhibiting a slight blue shift relative to the pure TTE solution, indicating elevated intermolecular interactions and possible confinement within salt‐rich domains (Figure 3a). Similarly, the interaction strengths of N70 and N71 in the respective electrolytes remain essentially unchanged compared to the pure substances, with only slight blue shifts of the Raman bands observed (Figure 3b).

Furthermore, the decrease in flammable organic species in the liquid electrolyte, achieved by incorporating the non‐flammable diluents, enhances the safety of the corresponding electrolyte formulations. Although TTE is often claimed to be non‐flammable, slight flammability is observed. In contrast, neither of the new diluents can be ignited. As a result, the flammability of the 7000, 7100, and 7071 can be reduced by roughly 40 % in contrast to BE, as revealed via self‐extinguishing time (SET) experiments (Figures 3c and S1).

Additionally, the viscosity can be reduced to 4.3 mPa∙s by adding N70, corresponding to a ≈19% improvement over BE. Because of the higher viscosity of N71 relative to N70, its presence in the liquid electrolyte increases the overall viscosity relative to 7000; however, it remains lower than the baseline formulation (Figure S2).

As ionic conductivity is directly linked to viscosity, particularly in moderately concentrated electrolytes and LHCEs, where ions are transferred via a vehicle‐type mechanism [21], the addition of the highly fluid diluents leads to an increased ionic conductivity. By combining N70 and N71 in 7071, the ionic conductivity can be further increased up to 2.2 mS cm^−1^, followed by 7000 and 7100, corresponding to an improvement of up to ≈20 % over the BE's ionic conductivity (Figure 3d). Further PFG‐NMR experiments confirm the increased diffusivity of all electrolyte components, including the desired Li^+^ ion diffusion (Figure 3e). Despite its higher viscosity, formulation 7071 (4.5 mPa∙s) exhibits higher ion diffusivity than 7000 (4.3 mPa∙s), deviating from the reference Walden plot (BE, 7000, 7100). This suggests that the combined presence of N70 and N71 alters Li^+^ ion clustering and the mesoscopic solvation structures, facilitating more efficient Li^+^ ion transport in 7071 (Figure S3) [31, 41].

**FIGURE 4:**
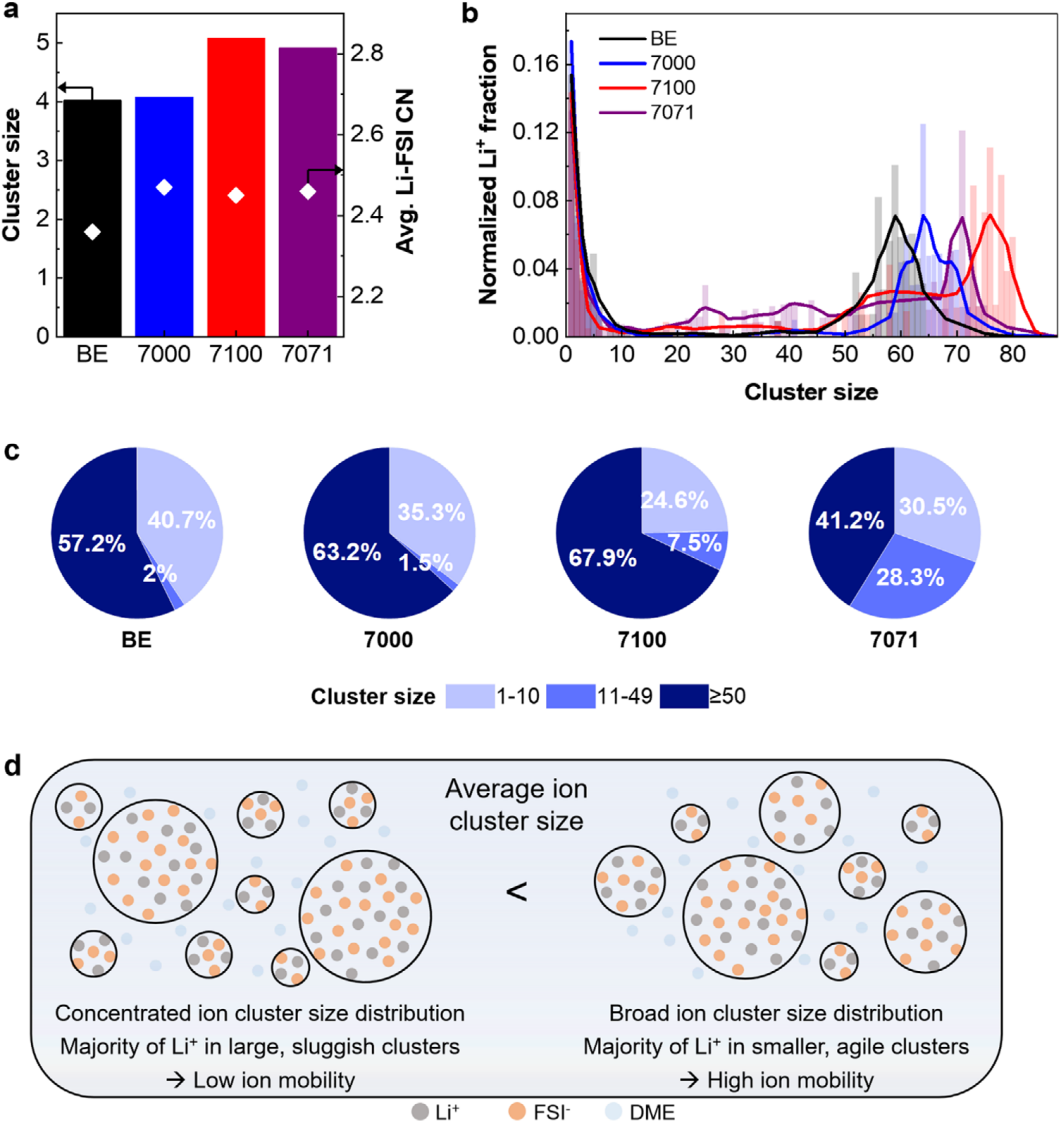
(a) Average Li^+^ ion cluster size and Li‐FSI coordination number (CN) via MD simulations at 300 K. (b) Cluster size distribution and (c) fraction of Li^+^ found in small (≤10 Li^+^ per cluster), medium‐sized (11 to 49 Li^+^ per cluster), and large clusters (≥50 Li^+^ per cluster) within the considered electrolyte formulations at 300 K. (d) Schematic showing the difference between an electrolyte formulation with a concentrated cluster size distribution, where the majority of Li^+^ ions is found in large ion clusters, which exhibits a low ion mobility even though the average cluster size is comparably low (right) and a liquid electrolyte with a broad ion cluster size distribution, where the majority of Li^+^ is found in smaller more agile cluster resulting in a high ion mobility despite its comparably higher average ion cluster size.

To further elucidate the effects of the new diluents on the solvation structure and ion mobility, molecular dynamics simulations at the mesoscopic scale were conducted. Consistent with the Raman results, a similar primary solvation environment of the Li^+^ ions is observed, as the population distribution of DME and FSI^−^ in lithium's first solvation shell largely remains with the majority of Li^+^ residing in FSI2DME1 and FSI1DME2 motifs (Figure S5). An average Li‐FSI coordination number of 2.36 in BE was determined, while 7000, 7100, and 7071 show slightly higher average values of 2.48, 2.46, and 2.46 (Figure 4a). Accordingly, all investigated electrolyte formulations feature anion‐rich Li^+^ solvation environments. Nonetheless, the data suggest a slightly enhanced Li^+^‐FSI^−^ association in the presence of N70 and N71. The MD investigations further reveal that neither TTE nor N70 nor N71 shows notable interactions with Li^+^. To further quantify the interface between diluents and salt phase, a surface‐area matrix using Voronoi tessellation was constructed, where the average geometric contact area between each molecular pair is determined [42]. In 7071, TTE shares 14.8% of its interfacial area with DME and 9.5% with FSI^−^, resulting in an anion‐to‐solvent preference of 0.64. In contrast, N70 and N71 exhibit lower ratios of 0.43 and 0.48, respectively, indicating a lower affinity toward FSI^−^ in comparison to TTE (Table S7). TTE's greater affinity toward FSI^−^ rationalizes the comparably lower Li^+^‐FSI^−^ coordination number in BE as well as the experimentally observed phase behavior with N7X during electrolyte formulation. Concomitantly, the salt cluster interface is “fuzzier” for BE as compared to all other formulations, reflected by a larger total surface area (Figure S6).

In contrast to recent reports, the increase in molecular diversity resulting from the addition of N70 or N71 does not lead to the desired Li^+^ ion cluster size reduction, as BE exhibits the smallest average cluster size (4.0), while 7071, despite having the highest molecular diversity, shows the second‐highest average Li^+^ ion cluster size (4.9) after 7100 (Figure 4a) [31]. These observations are seemingly inconsistent with the observed elevated ion diffusivities in 7071, which are usually correlated with either reduced viscosity, smaller ion clusters, or reduced Li^+^‐anion interactions. Closer inspection of the formed clusters shows that, despite its larger average Li cluster size, 7071 exhibits a markedly broader cluster‐size distribution (Figure 4b). In BE, 7000 and 7100, the majority of the Li^+^ ions reside either in small (≤10 Li^+^ per cluster) or large clusters (≥50 Li^+^ per cluster) across the electrolyte formulations, while 7071 features a notable population of medium‐sized clusters (11 to 49 Li^+^ per cluster). Thus, while having the second highest average cluster size, only 41 % of Li^+^ ions in 7071 are found in large salt clusters, compared to 57 %, 63%, 67 % in BE, 7000, and 7100, respectively (Figure 4c). These large clusters are expected to exhibit low diffusivity and to play only a minor role in the overall ionic conductivity. Notably, at elevated temperatures (350 K), the distributions for all formulations broaden and become less concentrated, consistent with thermally accelerated cluster‐formation kinetics that reduce the dominance of large clusters (Figure S7b). Conversely, at low temperatures (250 K), the cluster size distribution shifts toward the formation of large Li^+^ ion clusters, which, in combination with increased viscosity, leads to reduced ionic conductivity below 20°C across all electrolyte formulations (Figure S7a). Notably, the magnitude of this effect highly depends on the electrolyte composition. 7071 shows the largest increase in the formation of large ion clusters and, correspondingly, the steepest ionic conductivity loss upon cooling (Figure S7c). As a consequence, below −10°C, 7000 becomes more ionically conductive than 7071, highlighting the highly composition‐ and temperature‐sensitive nature of the mesoscopic solvation landscape and its impact on bulk ion transport.

Overall, while the average cluster size is a useful metric, the identified highly dynamic cluster‐size distribution provides a more comprehensive measure, offering deeper insights into the mesoscopic solvation structure and correlated ion dynamics (Figure 4d).

### 2.3 Electrochemical Performance Evaluation in Lithium Metal‐Based Cells

**FIGURE 5:**
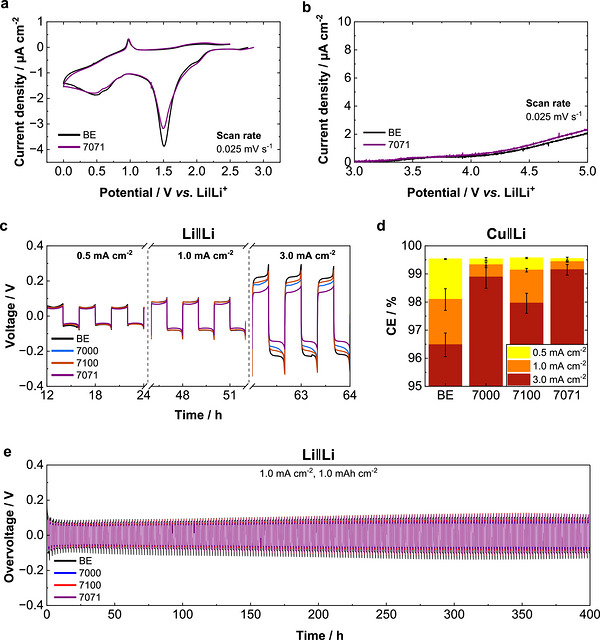
(a) Reductive cyclic voltammogram and (b) oxidative linear sweep voltammogram with and without the presence of the considered diluents. (c) Overvoltage evolution of symmetric LiǁLi cells and (d) average CE at three current densities (0.5, 1, 3 mA cm^−2^). (e) Overvoltage evolution in LiǁLi cells (1 mA cm^−2^, 1 mAh cm^−2^) with the evaluated liquid electrolyte formulations.

Cyclic voltammetry measurements show no additional decomposition peaks in the presence of N70 and N71, indicating that the diluents do not undergo redox decomposition within the electrochemical stability window of the reference electrolyte (Figure 5a,b). Additionally, the reductive current and position of the FSI^−^ anion reductive peaks (at ≈1.5 V vs. LiǀLi^+^) remain unchanged, further concluding the minor impact of the investigated diluents on the inner solvation structure of Li^+^ ions and the resulting reductive decomposition reactions during cell operation (Figure 5a).

To assess how the diluents affect the electrochemical performance of lithium metal‐based cells, the lithium stripping/plating reversibility was first evaluated in CuǁLi and symmetric LiǁLi cells (Figure 5c–e). At current densities of 0.5 mA cm^−2^, all considered electrolyte formulations promote a CE of 99.5 % and show no differences in the voltage profiles. However, after increasing the current density to 1.0 and 3.0 mA cm^−2^, differences between the different cell chemistries become apparent. In the cells with the baseline electrolyte, the overvoltages rise to ≈0.1 V at 1.0 mA cm^−2^ and exceed 0.3 V at 3.0 mA cm^−2^, while the CE drops to 98.1 % and 96.4 %, respectively. By contrast, the electrolyte formulations with N7X lead to comparably low increases in overvoltage and a lower decline in the CE at the elevated current densities (Figure 5c,d). The presence of the best‐performing liquid electrolyte (7071) in the resulting cell enables overvoltages of 0.15 V, while maintaining a CE >99 % even at a current density of 3 mA cm^−2^. Analogues to the ionic conductivities, 7000 exhibits the second‐best and 7100 the third‐best high‐rate performance, while still achieving CEs of >98 % at 3 mA cm^−2^ (Figure 5c). During long‐term cycling, all four electrolytes promote homogeneous Li stripping/plating for more than 400 h (Figure 5e).

**FIGURE 6:**
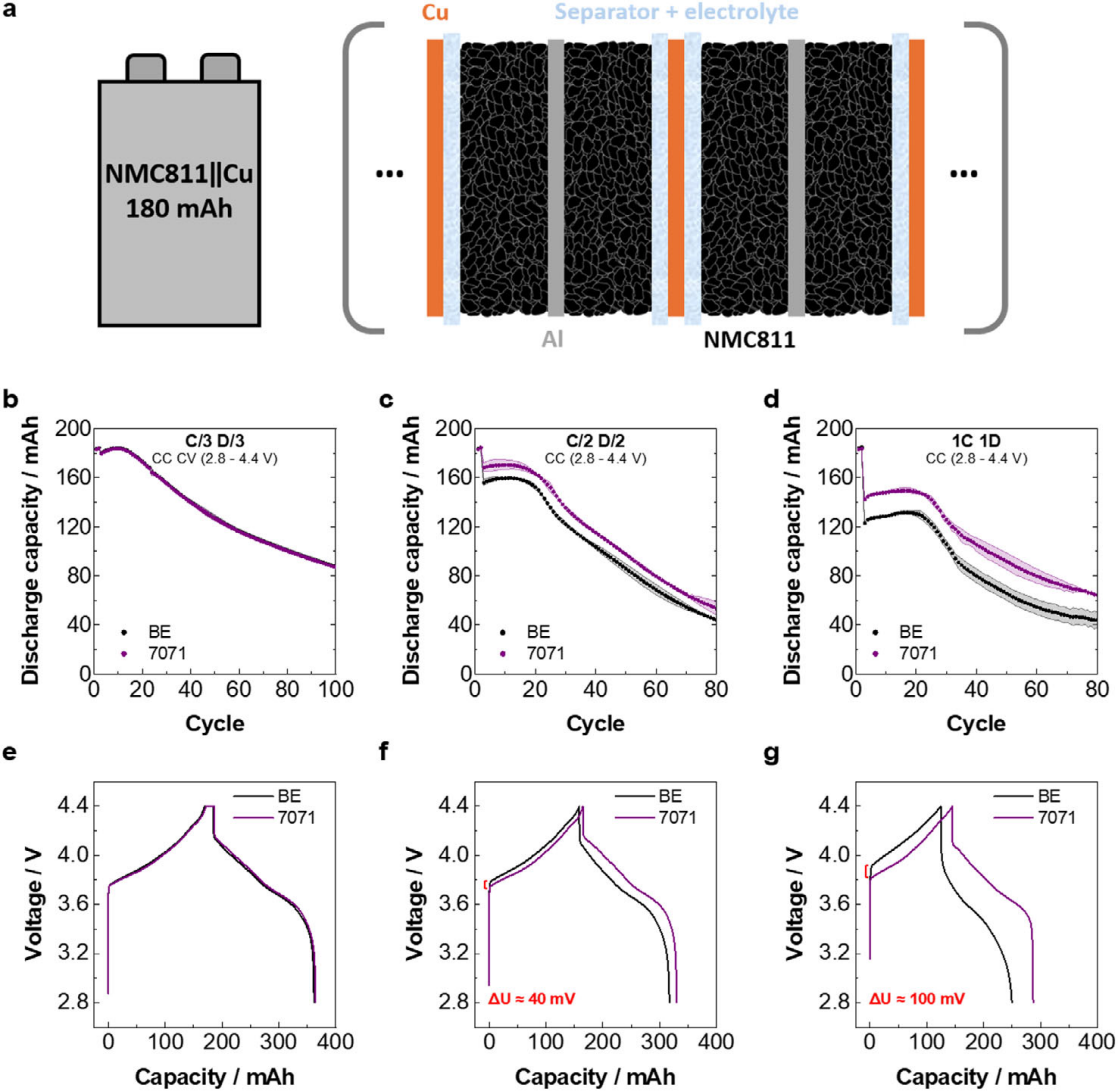
(a) Schematic illustration of the used zero‐excess NMC811ǁCu pouch cells (180 mAh) within this study. Discharge capacity evolution of zero‐excess NMC811ǁCu pouch cells with 2.5 g Ah^−1^ liquid electrolyte inventory at (b) C/3 D/3 (CC CV), (c) C/2 D/2 (CC), and (d) 1C 1D (CC) with two formation cycles at C/10 at the beginning of each experiment. Corresponding voltage profiles (fifth cycle) at (e) C/3 D/3 (CC CV), (f) C/2 D/2 (CC), and (g) 1C 1D (CC).

The best‐performing electrolyte formulation 7071 was further analyzed and compared with BE in a zero‐excess pouch cell under application‐oriented conditions (Figure 6a). Again, at low C‐rates and with the inclusion of a kinetically relaxing CV step during charge, a nearly identical evolution of the discharge capacity is observed with both electrolyte formulations, reaching the defined end‐of‐life at an 80 % state of health (SOH) after 40 cycles and surpassing the 100 mAh mark after 81 cycles (Figure 6b). However, under harsher charge/discharge conditions (C/2 and 1C) and with the elimination of the constant‐voltage (CV) charge step, a cycle life improvement with 7071 compared to cells with the baseline LHCE formulation is observed (Figure 6c,d). The cells with BE reach 80 % SOH and 100 mAh after 23 and 42 cycles at C/2 (Figure 6c), respectively. At 1C, the SOH drops below 80% after the two formation cycles, and the discharge capacity reaches 100 mAh after 29 cycles (Figure 6d). With 7071, the cycle life is extended to 48 cycles under the 1C high‐rate conditions before dropping below 100 mAh (Figure 6d). The origin can again be attributed to overvoltage during galvanostatic cycling, which reduces the accessible capacity. Under C/3 cycling conditions, the voltage profiles of the first cycle are nearly identical (Figure 6e); however, at higher C‐rates, the differences become increasingly pronounced. At C/2, the overvoltage difference between the two cell setups is ≈40 mV (Figure 6f), increasing to ≈100 mV at 1C (Figure 6g).

### 2.4 Interphase and Li Deposition Morphology Analysis

**FIGURE 7:**
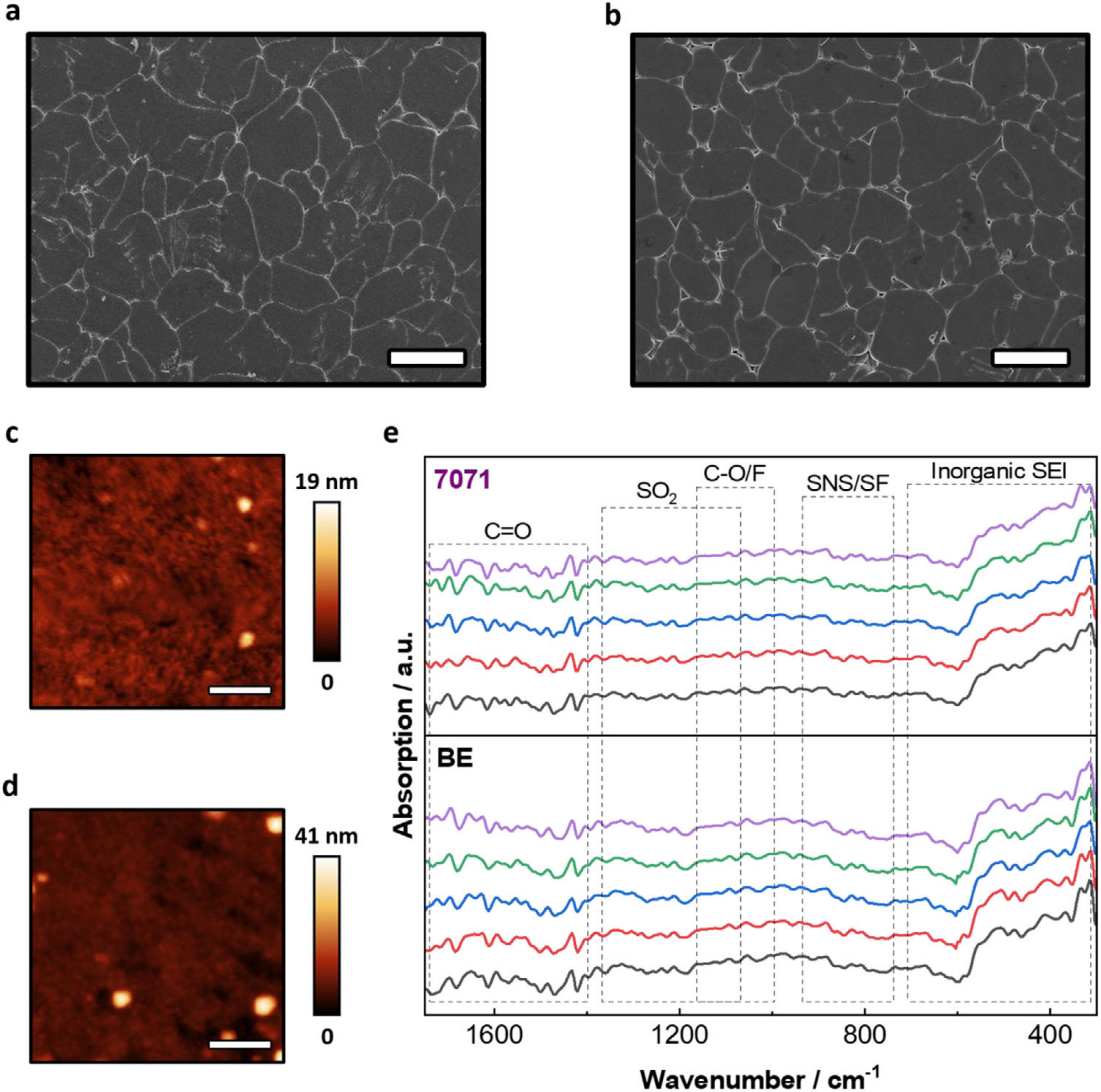
Li deposition morphology after plating of 5 mAh cm^−2^ Li on a copper electrode with (a) 7071 and (b) BE. AFM topography of the sputtered copper electrodes after polarization at 0.05 V versus LiǀLi^+^ with (c) 7071 and (d) BE. (e) Corresponding SINS absorption spectra of the performed line scans of the formed SEIs to investigate the deviations within and across the SEIs.

To investigate the impact of N70 and N71 on the electrode‐covering interphases and the Li deposition morphology, ex situ XPS and synchrotron‐assisted nanospectroscopy (SINS) [43, 44, 45], as well as experiments with a scanning electron microscope (SEM), were conducted. In both electrolyte formulations, Li deposits form a favorable, compact, mosaic‐like morphology after depositing 5 mAh cm^−2^ on the copper electrode. By preventing whisker‐like Li growth and reducing active surface area, such microstructures reduce continuous active material consumption and limit the formation of electrochemically isolated dead Li during subsequent Li electrodissolution [7]. These features correlate well with the high CEs and long cycle life observed in cells with the LHCE formulations, whose anion‐dominated solvation structure typically fosters more uniform Li deposits (Figure 7a,b) [19, 20].

Similar to the Li deposition morphology, SINS measurements revealed a structural and compositional homogeneity of the SEI with both electrolyte formulations (Figure 7c–e). Only a few round‐shaped artifacts cover the otherwise structurally homogeneous interphases, with an average roughness of 1–2 nm in both systems (Figure 7c,d). The compositional homogeneities were investigated via SINS line scans across the interphases, consisting of five independent measurements acquired across a 500 nm scan length (Figure S14). The compositions of the interphases are highly homogeneous, as the positions and intensities of the infrared (IR) absorption features in both spectra are consistent within each SEI (Figure 7e). Furthermore, across both interphases, nearly identical IR absorption is observed, with lower wavenumber bands associated with inorganic SEI components such as LiF and Li_2_O. Less absorption is observed in the higher wavenumber region, which can be mainly attributed to larger fragments of the FSI^−^ anion based on the S─F/S─N─S as well as SO_2_ stretching features in the range of 1400 to 800 cm^−1^ [46]. Pronounced bands are also observed above 1400 cm^−1^, which can be attributed to C═O vibrations, indicating the formation of carboxylates from solvent decomposition (Figure 7e) [14, 47, 48]. The round artifacts scattered across the SEI are identified as dried‐out electrolyte residue, exhibiting signatures similar to those of the dried formulations (Figure S15). The harvested electrodes were intentionally not washed to keep the SEI, particularly the organic components, as pristine as possible.

**FIGURE 8:**
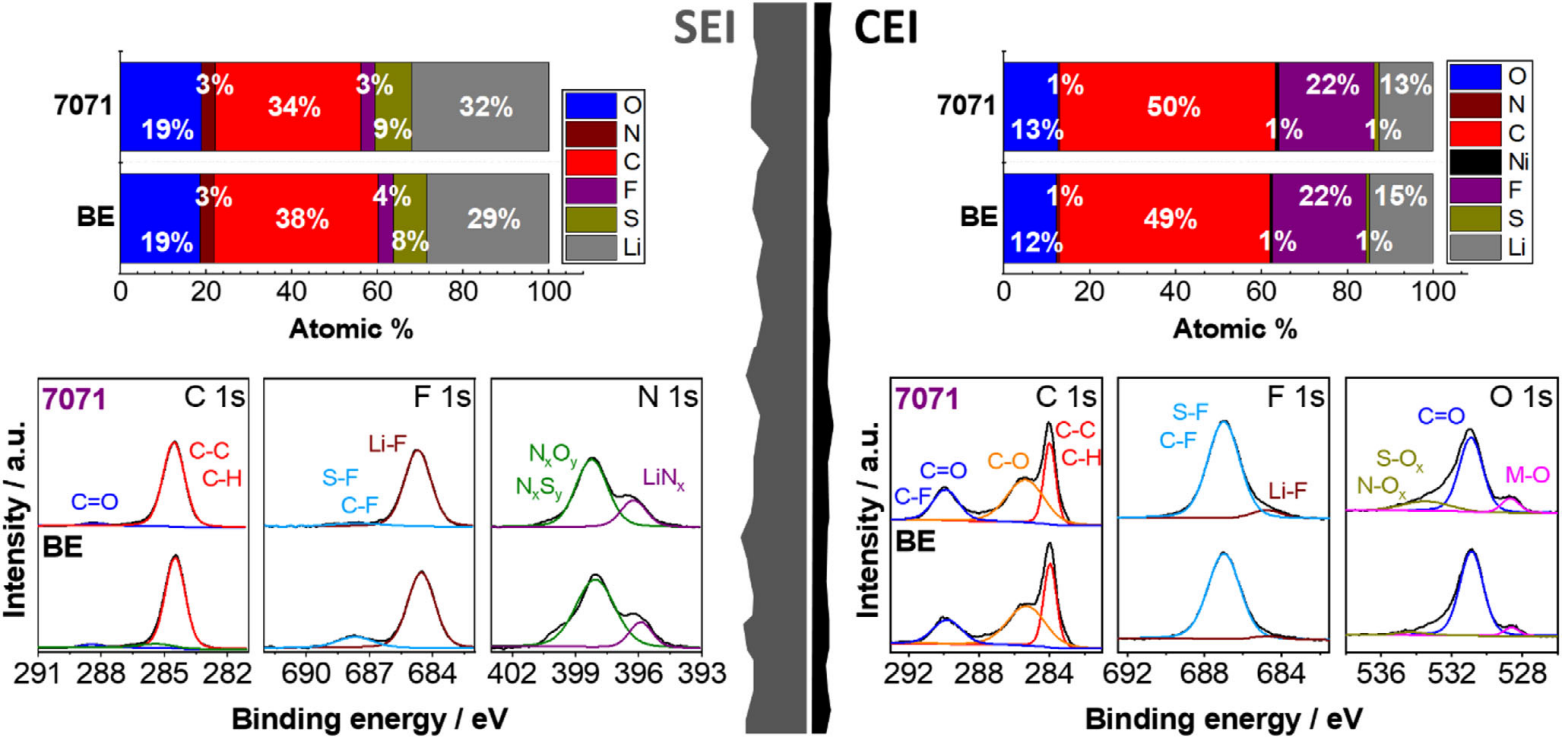
XPS analysis of the SEI (left) and CEI (right) with the elemental composition of the formed interphases (top) and the C1s, F1s, N1s (SEI only) and O1s (CEI only) core spectra (bottom). The Li metal and NMC811 electrodes were harvested after 20 charge/discharge cycles at 1C between 2.8 and 4.4 V.

Additional XPS measurements of the SEIs reveal no distinct differences between the two interphases and indicate similar compositions. Both interphase layers primarily consist of FSI^−^‐derived decomposition products, as evident in the F1s and N1s core spectra, as well as hydrocarbon compounds, based on the C 1s core spectra [14, 20, 31, 49]. As solely surface measurements were conducted without sputtering, the organic compounds, which are commonly found in the outer SEI, are relatively overrepresented. Similar to the SEI, the interphase on the positive electrode side, noted as CEI, shows no pronounced differences. Here, in addition to the binder (hydrofluorocarbon‐based), decomposition products such as Li_2_CO_3_ and LiF are observed (Figure 8) [50, 51].

In summary, the ex situ interphase analysis indicates that the addition of N70 and N71 does not interfere with anion‐derived interphase formation, either on the Li metal or on the NMC electrode side, leading to similar morphological and compositional interphases that promote a compact Li deposition morphology.

### 2.5 Decoupling Bulk Ion Transport From Interfacial Kinetics

**FIGURE 9:**
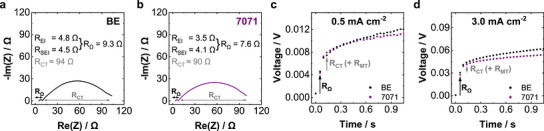
Nyquist plot obtained by EIS measurements (1 MHz to 1 mHz) of symmetric LiǁLi cells with (a) BE and (b) 7071. Voltage evolution of symmetric LiǁLi cells after applying (c) 0.5 mA cm^−2^ and (d) 3.0 mA cm^−2^. All of the measurements were conducted after 10 pre‐conditioning cycles (1.0 mA cm^−2^, 1.0 mAh cm^−2^) with R_El_: electrolyte resistance, R_SEI_: SEI resistance, R_Ω_: Ohmic resistance, R_CT_: charge transfer resistance, R_MT_: mass transport resistance.

To further substantiate that the observed high‐rate improvements with 7071 arise from enhanced bulk ion mobility and to rule out any interface contributions (e.g., Li^+^ ion desolvation), complementary electrochemical impedance spectroscopy (EIS) and intermittent galvanostatic cycling experiments in symmetric LiǁLi cells were conducted. After 10 pre‐conditioning cycles, the EIS spectra show similar contributions of the SEI resistance (>100 Hz, first semi‐circle) and the charge‐transfer resistance at the interfaces (<100 Hz, second semi‐circle) for 7071 and BE, making the reduced electrolyte‐induced Ohmic resistance with 7071 the only difference between the two systems (Figure 9a,b) [52, 53].

The intermittent galvanostatic cycling experiments at relevant current densities corroborate these observations. After applying 0.5 mA cm^−2^, the initial voltage jump, associated with Ohmic resistances, as well as the subsequent voltage response over the next few hundred milliseconds, which is dominated by charge transfer processes, closely overlap for both cells (Figure 9c) [53, 54]. Differences emerge only at higher timeframes (>0.5 s), where the 7071‐based cell exhibits a lower voltage rise than the baseline cells, consistent with reduced mass transport‐related limitations in the electrolyte. At higher current densities, the onset of this voltage divergence shifts to earlier times, as the dominance of mass transport processes increases and the charge‐transfer resistance's contribution diminishes, as expected from the Butler‐Volmer equation (Figure 9d) [55].

Overall, these observations indicate that incorporating N70 and N71 into the baseline electrolyte is responsible for the improved high‐rate capability, while interphase‐related resistances and charge transfer kinetics remain essentially unchanged.

## 3 Conclusion

In this study, two previously unexplored fluorinated ethers (N70 and N71) are, for the first time, identified and systematically validated as co‐diluents for (LHCEs, establishing an electrolyte design strategy that reveals their previously unrecognized potential to considerably enhance the performance of Li metal‐based cells. The combination of a broad electrochemical stability window, low viscosity, and intrinsic non‐flammability enabled the formulation of a safer liquid electrolyte compared to baseline formulations, while promoting superior high‐rate performance. In particular, the cells containing the ternary diluent blend TTE/N70/N71 outperformed the TTE‐only baseline electrolyte counterpart, by reducing the overvoltage in symmetric LiǁLi cells at 3 mA cm^−2^ by ≈40 % and substantially extending the cycle life of application‐oriented zero‐excess NMC811ǁCu pouch cells (180 mAh) at high galvanostatic cycling rates (1C) by roughly 80 % before reaching 100 mAh.

The enhanced performance was attributed primarily to the suppression of elevated ion clustering and a broader cluster size distribution, rather than to further reductions in viscosity relative to binary diluent systems 7000 and 7100, thereby improving ion mobility. By balancing reduced viscosity with mitigation of excessive ion clustering, the 7071 formulation facilitated effective Li^+^ ion transport, thereby reducing concentration polarization in the bulk electrolyte and lowering interfacial overvoltages at high current densities. Ex situ SEM, XPS, and SINS analyses, along with additional EIS and intermittent cycling experiments, revealed nearly identical interphase properties regardless of the presence of the investigated diluents, indicating that galvanostatic cycling performance improvements arise predominantly from enhanced mass transport in the bulk electrolyte rather than from improved interface processes and SEI properties.

## Author Contributions

**Andrew Dopilka**: investigation, formal analysis. **Adil Aboobacker**: investigation, validation, writing – original draft, writing – review and editing, formal analysis. **Dominik Weintz**: conceptualization, investigation, writing – original draft, writing – review and editing, validation, formal analysis. **Uta Rodehorst**: investigation, formal analysis. **Martin Winter**: supervision, funding acquisition, writing – review and editing, resources. **Robert Kostecki**: supervision, writing – review and editing. **Diddo Diddens**: supervision, writing – review and editing, formal analysis. **Anne Hockmann**: investigation, formal analysis. **Felix Pfeiffer**: investigation, formal analysis. **Isidora Cekic‐Laskovic**: funding acquisition, writing – review and editing, project administration, supervision, resources. **Monika Schönhoff**: writing – review and editing, supervision. **Christian Wölke**: investigation, formal analysis.

## Conflicts of Interest

The authors declare no conflicts of interest.

## Supporting information



The authors have cited additional references within the Supporting Information [36, 42, 56, 57, 58, 59, 60, 61, 62, 63, 64, 65, 66, 67, 68]. **Supporting File 1**: anie73091‐sup‐0001‐SuppMat.docx

## Data Availability

The data that support the findings of this study are available from the corresponding author upon reasonable request.
