# Supplementary material for: Heat Transfer Fluids as Co‐Diluents in Localized High‐Concentration Electrolytes for High‐Rate Lithium Metal Batteries With Enhanced Safety
Source: Angew Chem Int Ed Engl. 2026 Jun 16;65(34):e3797296. doi: 10.1002/anie.3797296 (PMC13480748; doi:10.1002/anie.3797296)
Supplement: Supplementary file 1 [file ANIE-65-e3797296-s001.docx]

**Heat Transfer Fluids as Co-Diluents in Localized High-Concentration Electrolytes for High-Rate Lithium Metal Batteries with Enhanced Safety**

Dominik Weintz^a^, Adil Aboobacker^a^, Andrew Dopilka^b^, Felix Pfeiffer^a^, Anne Hockmann^c,d^, Christian Wölke^a^, Uta Rodehorst^e^, Monika Schönhoff^c^, Robert Kostecki^b^, Diddo Diddens^a^, Martin Winter^a,e^, Isidora Cekic-Laskovic^a^

*^a^ Forschungszentrum Jülich GmbH, Helmholtz-Institute Münster (IMD-4), Corrensstraße 48, 48149 Münster, Germany*

*^b^ Energy Technologies and Systems Division, Lawrence Berkeley National Laboratory, Berkeley, California 94720, United States*

*^c^ Institute of Physical Chemistry, University of Münster, Corrensstraße 28/30, 48149 Münster, Germany*

*^d^ International Graduate School for Battery Chemistry, Characterization, Analysis, Recycling, and Application (BACCARA), University of Münster, Corrensstraße 40, 48149 Münster, Germany*

*^e^ MEET Battery Research Center, University of Münster, Corrensstraße 46, 48149 Münster, Germany*

[*i.cekic-laskovic@fz-juelich.de](mailto:*i.cekic-laskovic@fz-juelich.de)

**Experimental Part**

a) Materials

**Table S1.** Electrolyte components used to formulate the considered electrolyte formulations.

| **Material** | **Purity / %** | **Manufacturer** |
| --- | --- | --- |
| LiFSI | >99.9* | E-Lyte |
| DME | >99.9* | E-Lyte |
| TTE | >99.9* | E-Lyte |
| Methyl heptafluoropropylether (NOVEC7000^TM^) | >99.9** | Sigma Aldrich |
| Nonafluorobutylether (NOVEC7100^TM^) | >99.9** | Sigma Aldrich |

*(H_2_O < 10 ppm) **(H_2_O < 20 ppm)

Reagents used to formulate the investigated liquid electrolyte are listed in Table S1. The pristine **Li metal electrodes** were prepared using in-house-built cutting and pressing devices, as described in our previous publications.^[1,2]^ Here, the Li rod was first cut with two tungsten wires and then pressed between two Mylar foils to the desired thickness of 200 µm. Copper and aluminum foils, used as working electrodes, were rinsed with ethanol and dried together with the Celgard 2325 (coin and pouch cells) and Whatman GF/D separators (Swagelok cells) at 60 °C under vacuum before transfer to an argon glovebox for storage.

The **copper thin-film electrodes** used for the SINS experiments were fabricated by sequential DC sputtering onto a quartz wafer substrate (University Wafer, U01-210714-1: Fused Silica JGS2). First, a thin layer of Ti (30 nm) was DC sputtered (3 × 3” TORUS Mag Keeper sputter guns) as an adhesion layer, followed by deposition of a 1.0 μm thick Cu metal layer with an RMS surface roughness of 1.3 nm.

b) Cell assembly

**Two-electrode coin cells (CR2032)** were assembled by placing a Celgard 2325 separator soaked with 16 µl liquid electrolyte between the Li metal and the selected counter electrode (Li, Cu, or NMC). The cells, including the cell stack, two spacers, and a spring, were then sealed using an automated Hosen crimper (7 kN for 5 s).

For CV and LSV measurements, **three-electrode Swagelok cells** were assembled with a copper (cathodic scan) or aluminum (anodic scan) working electrode and a lithium metal counter electrode separated by a Whatman separator wetted with 120 µl liquid electrolyte. A Li metal reference electrode was placed perpendicular to the main electrode stack and was separated by an additional Whatman separator wetted with 80 µl of electrolyte.

The **180 mAh multilayer pouch** cells (NMC811ǁCu) were purchased from LiFUN Technology Corp. Ltd., and dried overnight at 60 °C under reduced pressure. The areal capacity of the NMC811 was 2.5 mAh cm^-2,^ and the separator consisted of a 9 µm polyethylene and a 3 µm ceramic layer. After drying, the cells were filled with 2.5 g Ah^-1^ of the respective electrolyte formulation and then vacuum-sealed at 15 % ambient pressure using a GN-HS350V by Gelon Lib Corp., Ltd. The cells were clamped between two metal plates with insulating layers, and a force-sensitive screwing tool was used to ensure a constant, reproducible stack pressure of ≈1.8 bar during cycling. After two formation cycles at 0.1C, the cells were opened to release the formed gas and then resealed.

For SINS measurements, sputtered copper electrodes were used in **two-electrode pouch cells**, with a Li metal foil (13 mm diameter, 1.0 mm thickness, MTI Corporation) as the counter electrode and a Celgard 2325 separator (20 mm diameter) soaked with 16 µl of liquid electrolyte. The pouch cells were assembled from poly foil bags (Sigma-Aldrich, Z183385) measuring 5.5 cm by 6 cm. Ni tabs were used to provide electrical connections to the electrodes, and each cell was heat-sealed with adhesive polymer tape (MTI Corporation, EQ-PLiB-NTA4). After cell assembly, a clamp was used to apply continuous pressure to the single-layer pouch cells.

The coin, Swagelok, and single-layer pouch cells were assembled in an argon-filled glovebox (O_2_ and H_2_O < 0.1 ppm), while the filling and sealing of the multilayer pouch cells were done in a dry room atmosphere (dew point < -60 °C).

c) Raman spectroscopy

**Raman spectroscopy** was conducted using a confocal Raman microscope (Horiba Scientific, LabRAM HR evolution, air-cooled CCD detector) with a 600 line/mm grating. Sample excitation was performed using a green laser (λ = 532 nm, 19 mW, attenuated to 1.9 mW at the sample). A 50x long-working distance objective lens (Carl Zeiss Microscopy, working distance: 9.2 mm, numerical aperture: 0.5) was utilized to focus the laser onto the samples. Spectra acquisition was done throughout 25 s with five accumulations to enhance signal clarity. Instrument control and subsequent data processing were carried out with LabSpec 6.6.2.7 (Horiba Scientific), and the system's calibration was conducted before the measurements using the Raman shift of crystalline silicon at 520.7 cm^-1^.

d) Viscosity measurements

The **dynamic viscosities** of the diluents and the formulated liquid electrolytes were determined with a rolling-ball viscometer (DMA 4002, Anton Paar). In this method, a metallic ball travels through a tube filled with the respective electrolyte sample. Based on the terminal velocity as well as the densities of the ball and the sample, the viscosity of the liquid electrolyte can be extracted. The measurements were conducted at an angle of 70° and repeated at least twice for each sample.

e) Impedance and NMR spectroscopy measurements

For **impedance measurements** to determine ionic conductivity, 2 ml Eppendorf Safe-Lock Tubes were filled with 750 μL of electrolyte in an argon-filled glovebox. The measurements were carried out using in-house-developed electrodes on a Metrohm Autolab/M204 potentiostat/galvanostat over the frequency range of 50 Hz to 20,000 Hz. The samples were equilibrated by maintaining each temperature in a temperature chamber (Memmert TTC256, with 0.1 °C temperature setting accuracy) for 2 h before measurement. The ionic conductivity of the considered electrolytes was measured over the temperature range −30 °C to 20 °C in 10 °C increments. The impedance spectra were fitted using the Metrohm Nova software with the following fitting model: Rs(CPE − Rp) (constant phase element (CPE)). Cell constants were determined using a 0.01 M KCl solution at 20 °C, and the average of five measurements was taken.

Self-diffusion coefficients (D) were measured *via* **pulsed-field-gradient nuclear magnetic resonance (PFG-NMR)** spectroscopy using a stimulated-echo (PGSTE) sequence. For each nucleus and sample, the gradient pulse duration and maximum gradient amplitude were optimized. The diffusion time was set to 100 ms, and the experiment was repeated with a diffusion time of 50 ms to assess potential convection artifacts. Experiments were carried out on a 60 MHz spectrometer.

f) DFT and MD simulations

**Density functional theory (DFT)** calculations were performed using the Gaussian 16 software. Geometry optimizations were carried out using the **ωB97X-D** range-separated hybrid functional in combination with the **6-311+G(d,p)** basis set.^[3,4]^ Solvent effects were included using the polarizable continuum model (PCM**)** with a dielectric constant of ε = 7.4, representing an ether-like environment comparable to DME-rich LHCEs.^[5]^ This approach accounts for condensed-phase dielectric screening while avoiding explicit solvent molecules. Frontier molecular orbital energies were obtained from the optimized structures.

**Classical molecular dynamics (MD)** simulations were performed using GROMACS 2019**.**6.^[6]^ Force field parameters for TTE, N70, and N71 were generated using LigParGen, which provides OPLS-AA–compatible parameters.^[7,8]^ The CHELPG charges obtained from DFT calculations were used for all organic species. To account for electronic polarization effects in the condensed phase, the partial charges of Li⁺ and FSI⁻ ions were uniformly scaled by a factor of 0.8.^[9,10]^

For each electrolyte system, an annealing-based equilibration protocol was employed to ensure proper structural relaxation. The steps to this protocol are:

(1) Energy minimization at 0 K,
 (2) equilibration at 300 K for 2 ns,
 (3) heating from 300 K to 450 K over 1 ns, followed by a 1 ns hold at 450 K, and
 (4) cooling back to 300 K over 1 ns and further equilibration at 300 K for 5 ns.

An analogous protocol was applied for simulations with an equilibrium temperature of 350 K. Following equilibration, production simulations were carried out for 20 ns in the NVT ensemble.

Initial simulation boxes were constructed by packing molecules according to the compositions listed in Table S2 using Packmol.^[11]^

**Table S2.** Composition of the simulation boxes for the investigated electrolyte formulations.

| **Electrolyte** | **LiFSI** | **DME** | **TTE** | **N70** | **N71** |
| --- | --- | --- | --- | --- | --- |
| BE | 100 | 120 | 300 | - | - |
| 7000 | 100 | 120 | 133 | 167 | - |
| 7100 | 100 | 120 | 150 | - | 150 |
| 7071 | 100 | 120 | 133 | 83 | 83 |

Custom analysis scripts supported by MDAnalysis were used to characterize the coordination environment and salt clustering behavior.^[12]^ Trajectory frames were sampled every 250 ps.
For coordination analysis, the number and identities of unique coordinating species within a 2.5 Å cutoff of each Li⁺ ion were identified, which only includes species within the primary solvation shell. The coordination statistics were averaged over all Li⁺ ions and trajectory frames, and the results were visualized as heatmaps to highlight the dominant coordination environments.

Salt clusters were identified using a connected-components algorithm based on Li⁺–FSI⁻ connectivity. For each analyzed frame, clustering was initiated from an unvisited Li⁺ ion. All FSI⁻ anions with at least one oxygen atom within a cutoff distance of 2.5 Å from the Li⁺ ion were included in the cluster. Subsequently, all Li⁺ ions located within 2.5 Å of any oxygen atom of the included FSI⁻ anions were added to the same cluster and marked as visited. This iterative procedure was repeated, alternately adding FSI⁻ anions and Li⁺ ions that satisfy the cutoff criterion, until no additional ions could be included, at which point the cluster was considered complete. The algorithm then proceeded to the next unvisited Li⁺ ion.

Clustering was completed once all Li⁺ ions in the system had been visited, yielding a set of Li⁺ cluster sizes for each snapshot. Cluster size statistics were subsequently accumulated over frames and averaged over the equilibrated portion of the trajectory.

Salt cluster size distributions were analyzed using a Li-weighted definition, in which each cluster contributes proportionally to the number of Li⁺ ions it contains. The Li-weighted probability $P_{Li}\left( S \right)$ represents the fraction of Li⁺ ions belonging to clusters of size $S$ensuring that the distributions reflect the local environments experienced by Li⁺ rather than the population of clusters themselves. Distributions were normalized and accumulated over the equilibrated portion of the trajectories. Smoothed curves are shown solely as visual guides.
Further structural and interfacial analyses were performed using TRAVIS^[13]^. Voronoi tessellation–based analysis was employed to quantify time-averaged geometrical contact areas between all molecular species. From the Voronoi construction, a matrix of average pairwise contact areas was obtained, where each element represents the mean contact area per molecule between two species.

To assess the participation of different diluent molecules in the salt interphase, the Voronoi contact-area matrix was analyzed for each diluent species. For a given diluent component, its total surface area was decomposed into contributions arising from contacts with salt species (Li⁺ and FSI⁻), coordinating solvent (DME), and other diluent molecules. Ratios of diluent–FSI to diluent–DME contact areas were used as an area-based measure of interfacial compositions, reflecting the extent to which anions directly participate in the diluent–salt interface.^[14]^

In addition, the average interfacial contact area per diluent molecule with the salt domain was computed by summing the Voronoi contact areas between each diluent molecule and all species belonging to the salt-conducting domain, defined as Li⁺, FSI⁻, and DME. This metric provides a size-agnostic measure of the degree to which different diluent molecules participate in the salt interphase.

g) Flammability test

The flammability of the electrolyte was determined using a **self-extinguishing time (SET) measurement** according to the following protocol.^[15,16]^ Five Whatman separators (Ø12 mm) were attached to a metallic needle, soaked in 0.4 ml electrolyte, and exposed to the flame of a universal lighter for 10 s. After ignition, the time was recorded until the flame extinguished. The experiment was repeated five times to ensure reproducibility, and the SET was then normalized to the sample mass.

h) Electrochemical measurements

**Galvanostatic cycling performance** was evaluated on a Maccor Series 4000 battery cycler at 20 °C using the specified currents and C-rates. The average CE in CuǁLi cells was measured using a Li reservoir protocol, where, after an initial formation cycle (5 mAh cm^-2^ Li metal deposition onto Cu and complete stripping until 1 V), 5 mAh cm^-2^ of Li metal was deposited on the copper electrode to serve as a reservoir. Then, 10 plating/stripping cycles at 0.5, 1, or 3 mA cm^-2^, each with an areal capacity of 1 mAh cm^-2^. Finally, the remaining Li reservoir was completely stripped to 1 V, and the average CE was calculated as the total stripping capacity divided by the total plating capacity (excluding formation cycle).

The NMC811ǁCu cell cycling experiments were performed with zero-excess LiFUN pouch cells (180 mAh), which were filled with 500 mg of the considered liquid electrolyte and then sealed inside the dry room.

**Cyclic voltammetry (CV)** and **linear sweep voltammetry (LSV)** measurements were conducted in a three-electrode cell with an additional Li metal electrode used as the reference, at 20 °C and a scan rate of 0.025 mV s^-1^.

The **EIS** and **intermittent galvanostatic cycling** experiments were conducted in a symmetric LiǁLi coin cell setup at 20 °C, following 10 preconditioning cycles (1 mA cm^-2^, 1 mAh cm^-2^). The experiments (EIS, CV, LSV, intermittent galvanostatic cycling) were done on a BioLogic VMP3, and the impedance was recorded over a frequency range of 1 MHz to 1 mHz.

i) X-ray photoelectron spectroscopy

**XPS** signals were collected on an Axis Ultra DLD spectrometer by Kratos Analytical with monochromatic Al Kα X-rays (hν = 1486.6 eV) at an emission angle of 0° and an accelerating voltage of 12 kV. The pressure was maintained at 1·10^-7^ mbar during the experiment and at least three measurements across two electrodes were conducted to ensure reproducibility. NMC811ǁLi coin cells were galvanostatically cycled for 50 cycles at 1C (1 mA cm^−2^), the retrieved electrodes were washed with 1 ml of DME, and transferred from the glovebox to the XPS in a sealed, argon-filled transfer container to avoid contact with air. The samples were characterized without sputtering, and the data were fitted with CasaXPS, including an energy scale calibration using the LiF peak at 684 eV.

j) Synchrotron infrared nanospectroscopy

**Synchrotron infrared nanospectroscopy (SINS)** measurements were performed with a Neaspec neaSCOPE microscope at the advanced light source (ALS), beamline 2.4, using PtIr-coated tapping probes (Neaspec). The microscope was operated inside a nitrogen-purged environmental chamber (O_2_ < 20 ppm). AFM topography images were acquired in tapping mode using a tapping amplitude of 50–60 nm. Images and the root-mean-square (RMS) surface roughness were calculated in Gwyddion.

SINS spectra were recorded under the same tapping conditions with a spectral resolution of 8 cm^–1^. Near-field “whitelight” IR nanoimages were collected concurrently with topography by fixing the interferometer mirror position to the most intense feature of the interferogram and collecting the second harmonic near-field amplitude value at each pixel. Reference spectra were taken on a polished Au wafer. Spectra were processed using neaPLOT (neaspec). All spectra were demodulated at the n = 2 harmonic. In this work, strong resonances characterized by substantial phase responses were observed; therefore, the second-harmonic imaginary component of the SINS signal was normalized to Au using IR absorption. This is because, in these strongly resonant cases, the imaginary part most closely matches the IR absorption data in the databases.

**Supplementary data**

**Table S3.** Physicochemical properties of the considered diluents.

| **Property** | **Methoxyhepta-fluoropropane (N70)** | **Methyl nonafluoro-butylether (N71)** | **1,1,2,2-Tetrafluoroethyl 2,2,3,3-tetrafluoropropylether (TTE)** |
| --- | --- | --- | --- |
| Molecular weight / g mol^-1^ | 200 | 250 | 350 |
| Boiling point / °C | 34 | 61 | 98 |
| Freeze point / °C | -122 | -135 | -38 |
| Flash point / °C | - | - | 27 |

**Table S4.** Composition of the considered electrolyte formulations for this study.

| **Abbreviation** | **Electrolyte formulation** |
| --- | --- |
| BE | LiFSI in DME/TTE (1:1.2:3 n/n) |
| 7000 | LiFSI in DME/N70/TTE (1:1.2:1.66:1.33 n/n) |
| 7100 | LiFSI in DME/N71/TTE (1:1.2:1.5:1.5 n/n) |
| 7071 | LiFSI in DME/N70/N71/TTE (1:1.2:0.83:0.83:1.33 n/n) |

**Table S5.** Intrinsic physicochemical properties of the investigated electrolyte formulations.

| **Electrolyte** | **Viscosity / mPa∙s** | **Density / g ml^-1^** | **Ionic conductivity / mS cm^-1^** | **SET / s g^-1^** |
| --- | --- | --- | --- | --- |
| BE | 5.177 | 1.498 | 1.87 | 37 ± 3 |
| 7000 | 4.344 | 1.467 | 2.00 | 23 ± 3 |
| 7100 | 4.758 | 1.492 | 1.94 | 22 ± 3 |
| 7071 | 4.513 | 1.481 | 2.22 | 23 ± 3 |

**Figure S1.** Time-resolved image sequences of the conducted SET determination measurements with considered electrolytes.

**Figure S2**. Dynamic viscosities of the considered electrolyte formulations at 20 °C.

**Figure S3.** Walden plot of the investigated electrolyte formulations, with BE, 7000, and 7100 as references. η^−1^ refers to the fluidity and σ to the ionic conductivity.

**Figure S4**. Molecular dynamics snapshot depicting the solvation structure for all considered electrolyte formulations at 300 K.

**Figure S5**. Li coordination motif *via* MD simulations at 300 K for all considered electrolyte formulations.

**Figure S6.** Surface area of the salt clusters within the used liquid electrolytes determined *via* MD simulation.

**Figure S7.** Cluster size distribution *via* MD simulations at **(a)** 250 K and **(b)** 350 K. **(c)** Ionic conductivities of the considered electrolyte formulations from -30 to 20 °C.

**Table S6.** Mean contact area between each electrolyte component within 7071 in Å^2^.

|  | **N70** | **TTE** | **N71** | **FSI^-^** | **DME** | **Li^+^** | **Sum** |
| --- | --- | --- | --- | --- | --- | --- | --- |
| **N70** | 61.94 | 86.74 | 52.98 | 13.44 | 31.14 | 0.21 | 246.45 |
| **TTE** | 54.13 | 90.47 | 48.55 | 24.36 | 37.8 | 0.43 | 255.73 |
| **N71** | 52.98 | 77.79 | 43.84 | 14.13 | 29.17 | 0.28 | 218.19 |
| **FSI^-^** | 11.15 | 32.4 | 11.73 | 31.49 | 61.34 | 24.33 | 172.44 |
| **DME** | 21.54 | 41.89 | 20.18 | 51.11 | 42.47 | 15.97 | 193.16 |
| **Li^+^** | 0.17 | 0.57 | 0.23 | 24.33 | 19.16 | 0.02 | 44.49 |

**Table S7.** Normalized fraction of contact area between the diluents (TTE, N70, and N71) and the components within the salt phase (Li^+^, FSI^-^, DME) of 7071.

|  | **FSI^-^** | **DME** | **Li^+^** |
| --- | --- | --- | --- |
| **N70** | 5.45 | 12.64 | 0.09 |
| **TTE** | 9.53 | 14.78 | 0.17 |
| **N71** | 6.47 | 13.37 | 0.13 |

**Table S8**. Average Coulombic efficiencies based on the measurements in CuǁLi cells

(Li reservoir protocol).

| **Current density / mA cm^-2^** | **Electrolyte formulation** | **Coulombic efficiency / %** | | |
| --- | --- | --- | --- | --- |
| 0.5 | BE | 99.51 | 99.54 | 99.56 |
|  | 7000 | 99.54 | 99.47 | 99.58 |
|  | 7100 | 99.59 | 99.54 | 99.58 |
|  | 7071 | 99.60 | 99.53 | 99.50 |
| 1.0 | BE | 98.53 | 97.62 | 97.82 |
|  | 7000 | 99.21 | 99.36 | 99.40 |
|  | 7100 | 99.08 | 99.20 | 99.11 |
|  | 7071 | 99.42 | 99.43 | 99.45 |
| 3.0 | BE | 95.92 | 96.12 | 96.03 |
|  | 7000 | 99.21 | 99.01 | 98.88 |
|  | 7100 | 98.27 | 98.03 | 97.96 |
|  | 7071 | 99.19 | 98.94 | 99.14 |

**Figure S8. (a)** Voltage profiles of the Coulombic efficiency measurements at 0.5 mA cm^-2^ in CuǁLi cells using the Li reservoir protocol. **(b)** Zoomed-in view of the final stripping step.

**Figure S9. (a)** Voltage profiles of the Coulombic efficiency measurements at 1.0 mA cm^-2^ in CuǁLi cells using the Li reservoir protocol. **(b)** Zoomed-in view of the final stripping step.

**Figure S10. (a)** Voltage profiles of the Coulombic efficiency measurements at 3.0 mA cm^-2^ in CuǁLi cells using the Li reservoir protocol. **(b)** Zoomed-in view of the final stripping step.

**Figure S11**. Development of overvoltage profiles during Li plating/stripping experiments in symmetric LiǁLi cells.

**Figure S12.** Corresponding Coulombic efficiency evolution of zero-excess NMC811ǁCu pouch cells with 2.5 g Ah^-1^ liquid electrolyte at **(a)** C/3 D/3 (CC CV), **(b)** C/2 D/2 (CC), and **(c)** 1C 1D (CC) with two formation cycles at C/10 at the beginning of each experiment.

**Figure S13.** **(a)** AFM image of the sputtered Cu electrode (RMS roughness: 1.3 nm). **(b)** SINS reflection spectra of Au (reference) for the synchrotron light source (ALS BL 2.4).

Scale size: 500 nm

**Figure S14**. Whitelight images corresponding to the topography images, including measurement spots of the linescans for **(a)** BE and **(b)** 7071.

Scale size: 500 nm

**Figure S15**. Whitelight image **(a)** and **(b)** SINS spectra of a round-shaped artifact in the whitelight and topography images with BE.

Scale size: 500 nm

**Figure S16.** Selected high-resolution XPS core spectra (C 1s, F 1s, and O 1s) of the pristine NMC811 electrodes.

**References**

[1]	D. Weintz, S. P. Kühn, M. Winter, I. Cekic-Laskovic, “Tailoring the Preformed Solid Electrolyte Interphase in Lithium Metal Batteries: Impact of Fluoroethylene Carbonate” *ACS Appl. Mater. Interfaces* **2023**, *15*, 53526–53532.

[2]	S. P. Kühn, F. Pfeiffer, M. Bela, U. Rodehorst, D. Weintz, M. Stan, M. Baghernejad, M. Winter, I. Cekic-Laskovic, “Back to the basics: Advanced understanding of the as-defined solid electrolyte interphase on lithium metal electrodes” *J. Power Sources* **2022**, *549*, 232118.

[3]	J.-D. Chai, M. Head-Gordon, “Long-range corrected hybrid density functionals with damped atom–atom dispersion corrections” *Phys. Chem. Chem. Phys.* **2008**, *10*, 6615.

[4]	R. Krishnan, J. S. Binkley, R. Seeger, J. A. Pople, “Self-consistent molecular orbital methods. XX. A basis set for correlated wave functions” *J. Chem. Phys.* **1980**, *72*, 650–654.

[5]	D. M. York, M. Karplus, “A Smooth Solvation Potential Based on the Conductor-Like Screening Model” *J. Phys. Chem. A* **1999**, *103*, 11060–11079.

[6]	M. J. Abraham, T. Murtola, R. Schulz, S. Páll, J. C. Smith, B. Hess, E. Lindahl, “GROMACS: High performance molecular simulations through multi-level parallelism from laptops to supercomputers” *SoftwareX* **2015**, *1–2*, 19–25.

[7]	L. S. Dodda, I. Cabeza de Vaca, J. Tirado-Rives, W. L. Jorgensen, “LigParGen web server: an automatic OPLS-AA parameter generator for organic ligands” *Nucleic Acids Res.* **2017**, *45*, W331–W336.

[8]	W. L. Jorgensen, D. S. Maxwell, J. Tirado-Rives, “Development and Testing of the OPLS All-Atom Force Field on Conformational Energetics and Properties of Organic Liquids” *J. Am. Chem. Soc.* **1996**, *118*, 11225–11236.

[9]	C. M. Breneman, K. B. Wiberg, “Determining atom‐centered monopoles from molecular electrostatic potentials. The need for high sampling density in formamide conformational analysis” *J. Comput. Chem.* **1990**, *11*, 361–373.

[10]	M. Maiti, A. N. Krishnamoorthy, Y. Mabrouk, N. Mozhzhukhina, A. Matic, D. Diddens, A. Heuer, “Mechanistic understanding of the correlation between structure and dynamics of liquid carbonate electrolytes: impact of polarization” *Phys. Chem. Chem. Phys.* **2023**, *25*, 20350–20364.

[11]	L. Martínez, R. Andrade, E. G. Birgin, J. M. Martínez, “P ACKMOL : A package for building initial configurations for molecular dynamics simulations” *J. Comput. Chem.* **2009**, *30*, 2157–2164.

[12]	N. Michaud‐Agrawal, E. J. Denning, T. B. Woolf, O. Beckstein, “MDAnalysis: A toolkit for the analysis of molecular dynamics simulations” *J. Comput. Chem.* **2011**, *32*, 2319–2327.

[13]	M. Brehm, M. Thomas, S. Gehrke, B. Kirchner, “TRAVIS—A free analyzer for trajectories from molecular simulation” *J. Chem. Phys.* **2020**, *152*, 164105.

[14]	A. Hockmann, M. Schönhoff, D. Diddens, “Analyzing the internal interface in localized high-concentration electrolytes” *J. Chem. Phys.* **2025**, *163*, 084719.

[15]	S. Hess, M. Wohlfahrt-Mehrens, M. Wachtler, “Flammability of Li-Ion Battery Electrolytes: Flash Point and Self-Extinguishing Time Measurements” *J. Electrochem. Soc.* **2015**, *162*, A3084–A3097.

[16]	Q. Li, Y. Xu, Y. Pan, W. Wang, G. H. Yeoh, “Review and Future Perspectives on Lithium Battery Fire Safety: Focusing on Design of Organic Components” *ENERGY Environ. Mater.* **2025**, *8*, e12892.
